# Considerations in selecting postoperative analgesia for pregnant sheep following fetal instrumentation surgery

**DOI:** 10.1093/af/vfz019

**Published:** 2019-06-25

**Authors:** Tamara J Varcoe, Jack R T Darby, Kathryn L Gatford, Stacey L Holman, Pearl Cheung, Mary J Berry, Michael D Wiese, Janna L Morrison

**Affiliations:** 1 Early Origins of Adult Health Research Group, University of South Australia, Adelaide, SA, Australia; 2 School of Pharmacy and Medical Sciences, University of South Australia, Adelaide, SA, Australia; 3 Robinson Research Institute, Adelaide Medical School, University of Adelaide, Adelaide, SA, Australia; 4 Department of Paediatrics and Child Health and Centre for Translational Physiology, University of Otago, Wellington, New Zealand

**Keywords:** analgesia, fetus, pregnancy, sheep, surgery

**Implications**

- As a model of human pregnancy, research studies in catheterized fetal sheep have made significant contributions to our understanding of both normal and abnormal fetal development.
- Owing to the invasive nature of catheterization surgery, effective postoperative analgesia is required.
- Decisions regarding appropriate analgesia should consider effectiveness in reducing maternal pain and fetal well-being as well as potential impacts upon fetal physiological parameters under investigation.
- The reporting of analgesic regimes in all research studies should comply with the ARRIVE guidelines.

## Sheep as a Model of Human Pregnancy

Studies in sheep have made significant contributions to our understanding of normal and abnormal fetal development, and have enabled scientists and clinicians to answer questions about the etiology and treatment of poor maternal, placental, and fetal health (Morrison et al., 2018). One fundamental advantage of utilizing sheep as an animal model of human pregnancy is the ability to surgically implant a diverse range of physiological monitoring systems into both maternal and fetal compartments. Implanting indwelling catheters into the maternal and fetal vasculature (Meschia et al., 1965) enables repetitive materno-fetal blood sampling free from any confounding effects of maternal, and therefore fetal anesthesia. In addition, surgical manipulation of the ovine fetus has provided key foundational knowledge for novel in utero treatment options for conditions such as spina bifida and diaphragmatic hernia. Incredibly rich information on fetal well-being can be captured from a highly dynamic in utero environment under a variety of experimental conditions that cannot be achieved from studies of a human fetus.

While pregnant ewes appear tolerant of abdominal/intrauterine surgery to access the fetus, effective postoperative analgesia is required both from an animal welfare perspective and to reduce the potential confounding effects of pain on the physiological variables under investigation. The effectiveness of postoperative analgesic regimens largely focus on maternal recovery. Whether or not the fetus is sensate, and therefore capable of experiencing pain as we understand it, is difficult to quantify and requires consideration of the physical, emotional, and cognitive aspects of pain perception. The fetus itself may be relatively less perceptive of postsurgical pain than its mother due to the physiological suppression of central nervous system activity by allopregnalone, a metabolite of progesterone, which maintains the fetus in sleeping behavioral states (Hirst et al., 2014; Mellor et al., 2005). Although the fetus may respond to painful stimuli, these reactions appear to be mediated via brainstem and subcortical pathways, and do not involve consciousness or awareness of pain (Mellor et al., 2005). Despite the importance of effective analgesia following surgery to instrument the fetus, we have observed that the type of drug, route of administration, and dosing schedule are often not reported.

In this review, we summarize the most commonly reported analgesics in sheep fetal vascular catheterization surgery (Table 1), discuss potential fetal impacts of each drug, and report original research conducted in our laboratory comparing the behavioral response to surgery following administration of two commonly used analgesics. We also provide a discussion of relevant considerations to be made when deciding upon an analgesic regimen for postsurgery analgesia. Importantly, we limit our discussion to systemic maternal administration, which potentially impacts the fetus as well as the mother. Although local or regional analgesia to maternal surgical wounds is also commonplace, it is beyond the scope of this review.

**Table 1.** Dosing regimen for analgesics commonly used systemically in studies utilizing maternal and fetal catheter surgery

Analgesic	Dose	Route of maternal administration	Dosing schedule	Mechanism of action	Potential fetal impacts	References
Buprenorphine	10–60 mg kg^−1^	Subcutaneous	Twice a day for 2 d	µ-Opiod receptor agonist, κ-opiod receptor antagonist	Untested	Limesand and Hay (2003), Jonker et al. (2008), Loughran et al. (2017)
Carprofen	1.4 mg kg^−1^	Intramuscular	Day of surgery and repeated daily as required based on evidence of pain.	Non-specific COX inhibitor	Untested, although possible impacts upon ductus arteriosus patency	Allison et al. (2016), Cleal et al. (2017), Shaw et al. (2018)
Flunixin meglumine	2.2 mg kg^−1^	Intramuscular	Day of surgery and repeated daily as required based on evidence of pain.	Non-specific COX inhibitor	Untested, although possible impacts upon ductus arteriosus patency	(Owens et al. (2007), Chang et al. (2016), Benjamin et al. (2017)
Meloxicam	0.5 mg kg^−1^	Subcutaneous	Day prior to surgery and repeated daily as required based on evidence of pain.	Selective COX-2 inhibitor	No impact upon fetal hemodynamics or COX expression in fetal tissues	Rac et al. (2006), Rac et al. (2007)
Xylazine	50 μg kg^−1^	Intramuscular	Upon awakening from anesthesia.	α2-adrenergic receptor	Increases myometrial activity, transiently suspends fetal breathing and decreases fetal heart rate and PaO_2_	Jansen et al. (1984), Darby et al. (2018), Soo et al. (2018)

## Commonly Used Analgesics for Sheep Fetal Catheterization Surgery

### Xylazine

Xylazine has been widely used in veterinary medicine and preclinical research studies to induce sedation or as an adjunct to injectable and inhalational anesthetics (Lemke, 2004). Xylazine is also effective in reducing experimentally induced acute, peripheral pain in sheep (Nolan et al., 1987; Grant et al., 1996), and has been used for postoperative maternal analgesia following fetal catheterization surgery, including within our own laboratory (Soo et al., 2017; Darby et al., 2018). Xylazine is an α2-adrenergic receptor agonist (Schwartz and Clark, 1998) for which a significant proportion of the antinociceptive effects are mediated via heteroceptors located in the dorsal horn of the spinal cord (Kyles et al., 1993). However, xylazine has a relatively short half-life of 22 min (Garcia-Villar et al., 1981), and bolus intravenous administration to an adult sheep provides analgesia to mechanical and thermal stimuli for approximately 60 min (Nolan et al., 1987). There are also well-documented side effects following the use of α2-adrenergic receptor agonists in a range of species, including hypoxemia, bradycardia, and hypotension (reviewed by Kästner, 2006). These effects occur at the higher doses required for sedation and anesthesia, and may not impact hemodynamic or cardiovascular measures at the lower doses used for analgesia (Grant and Upton, 2001). Nevertheless, the potential adverse consequences combined with a lack of sustained analgesic effects raise concerns about the appropriateness of xylazine as a postoperative analgesic.

### Buprenorphine

Buprenorphine is a potent analgesic commonly used to alleviate pain in research animals (Nolan et al., 1987), and has also been used as a maternal analgesic following fetal sheep catheter surgery (Loughran et al., 2017). As a partial µ-opioid agonist, buprenorphine hyperpolarizes centrally located neurons by opening K^+^ and closing Ca^2+^ channels, thus providing antinociceptive effects for thermal stimuli for up to three and a half hours (Waterman et al., 1991). However, the analgesic effect following mechanical noxious stimuli in sheep is variable (Waterman et al., 1991). Following a surgical insult in lambs, buprenorphine is effective in providing short-term analgesia as measured by behavioral responses to pain, but is less effective in reducing physiological responses such as cortisol secretion (Paull et al., 2008). Systemic administration to sheep has marked side effects, including agitation, chewing movements, bleating, rapid and frequent head movements, and respiratory depression (Nolan et al., 1987), and as a scheduled drug, the use, storage and administration of buprenorphine is heavily controlled.

### Non-steroidal anti-inflammatory drugs

Non-steroidal anti-inflammatory drugs (NSAIDs) are also routinely used for postoperative pain management in veterinary medicine (Lamont, 2008). NSAIDs inhibit both major isoforms of the cyclooxygenase (COX) enzyme (i.e., COX-1 and COX-2) and inhibit the processing of arachidonic acid to prostaglandins, thromboxanes, and leukotrienes (Vane and Botting, 1998), although relative inhibition of these isoforms varies between NSAIDs. The therapeutic effects largely result from their inhibition of prostaglandin production via the inducible COX-2 at sites of inflammation, whereas many of the side effects, such as gastrointestinal (GI) ulceration and bleeding, are attributed to inhibition of the constitutive COX-1 isoform (reviewed in Brune and Patrignani, 2015). Commonly used NSAIDs for maternal analgesia following fetal catheterization surgery include the relatively nonselective COX inhibitors carprofen (Allison et al., 2016) and flunixin meglumine (Owens et al., 2007). Similarly, the COX-2– selective NSAID meloxicam has demonstrated efficacy in reducing pain and inflammation following experimentally induced lameness (Colditz et al., 2019) and provides partial analgesia to lambs following ring castration (Paull et al., 2012). Due to its preferential inhibition of COX-2 over COX-1 (Engelhardt et al., 1996), meloxicam has a superior tolerance profile for upper GI tract effects in humans (Hawkey et al., 1998). Furthermore, unlike xylazine or buprenorphine, meloxicam does not cause respiratory suppression and can therefore be administered prior to surgery, thus ensuring plasma levels are at their highest at the time of the insult. With a half-life of 14 hr in nonpregnant sheep, the analgesic effects of meloxicam are sustained (Stock et al., 2013). However, NSAIDs are used with caution perioperatively in humans due to potential for renal dysfunction (Bell et al., 2018), although we are not aware of similar concerns in sheep.

Paracetamol administration has been given postoperatively to sheep recovering from fetal catheterization surgery (Malhotra et al., 2018). Although not an NSAID, paracetamol does suppress prostaglandin production by inhibition of COX. The analgesic properties of paracetamol, however, likely arise at multiple levels in the pain stimulus conduction pathway, including at tissue receptors, spinal cord, thalamus, and the cerebral cortex in which pain sensations are evoked (Jóźwiak-Bebenista and Nowak, 2014).

## Additional Considerations When Administering Analgesics During Pregnancy

**Figure 1.**
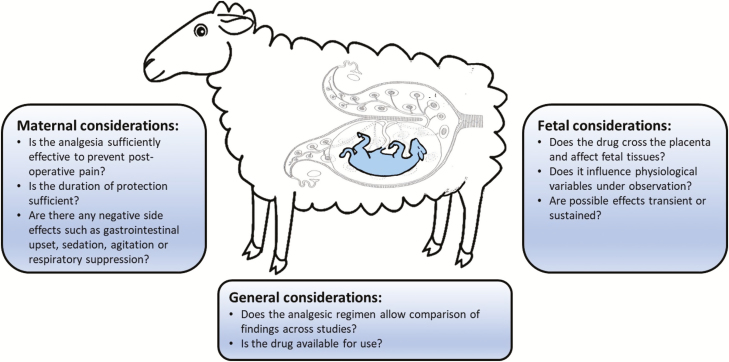
Considerations when selecting a postoperative analgesic regimen in studies of sheep fetal development.

Analgesic administration to pregnant ewes may have significant direct and indirect consequences for the fetus and may threaten the integrity and/or reproducibility of research outcomes. It is, therefore, important to consider both maternal and fetal effects of analgesia when deciding upon an analgesic regime (Figure 1).

Bolus xylazine administration to pregnant ewes increases myometrial activity, suspends fetal breathing, and decreases fetal heart rate and the partial pressure of oxygen (PaO_2_) (Jansen et al., 1984), although these effects are relatively transient and are unlikely to have long-term impacts upon the fetus. We are unaware of any studies examining the impact of maternal buprenorphine administration upon fetal lamb physiology; however, ex vivo studies of human term placenta suggest that there is low transplacental transfer of buprenorphine to the fetus (Nanovskaya et al., 2002).

Despite their widespread use and well-documented tolerability in nonpregnant animals, there are major concerns around the use of NSAIDs during pregnancy. Patency of the fetal ductus arteriosus (key to normal fetal cardiorespiratory status) is maintained by circulating prostaglandins derived from the placenta and by the low oxygen tension normally present in the fetus (Coceani and Olley, 1988). In humans, it is recommended that NSAIDs are not used in the third trimester of pregnancy (Walker and Misan, 1997) due to inhibition of prostaglandin synthesis increasing the risk of fetal, rather than postnatal, closure of the ductus arteriosus (Koren et al., 2006). Whether postoperative NSAID administration to sheep, in the doses required for analgesia, has detrimental impacts upon ductus arteriosus patency is unknown. Both COX-1 and COX-2 are expressed in the fetal sheep ductus arteriosus (Clyman et al., 1999), and in vitro inhibition of either isoform can cause contraction (Clyman et al., 1999). Furthermore, infusion of selective COX-2 inhibitors directly into the fetal vasculature (at doses much greater than experienced following maternal analgesic administration) induces contraction of the ductus arteriosus (Takahashi et al., 2000). However, in a sheep model of preterm labor, maternal meloxicam administration does not affect fetal acid–base status, plasma osmolality, arterial pressure, heart rate, or regional blood flow to the fetal kidney, intestine, adrenal glands, or cotyledon (Rac et al., 2006). Maternal meloxicam administration also does not affect COX expression in fetal kidney, small intestines, lung, liver, or heart (Rac et al., 2007). These results clearly demonstrate that a consideration of fetal impacts is warranted when developing analgesic regimes for preclinical research.

## Recent Experience

Decisions about postoperative analgesic regimen must consider effectiveness, duration of pain relief, potential side effects, and fetal impacts. Owing to concerns around sustained effectiveness, we transitioned from the use of xylazine analgesia to a regime of subcutaneous administration of meloxicam on the day prior to and following surgery, for all maternal/fetal indwelling catheter procedures conducted in our laboratory from 2017 onwards. Here we retrospectively compare the behavioral impact of meloxicam with xylazine, as assessed by prospective documentation of postoperative clinical condition, including feed intake. Studies using xylazine analgesia (2013–2016) and those using meloxicam analgesia (2017–2018) were all conducted at the South Australian Health and Medical Research Institute (SAHMRI) Preclinical Imaging Research Facility (PIRL), in accordance with the Australian Code of Practice for the Care and Use of Animals for Scientific Purposes (National Health and Medical Research Council 2013), and with approval from appropriate local Animal Ethics Committees.

Merino ewes between 105 and 125 days of gestation (term = 150 ± 3 days of gestation) were housed individually in a 12:12 (light:dark) hr photoperiod with ad libitum access to water. Animals were daily fed 1 kg lucerne chaff (85% dry matter, metabolizable energy content = 8.3 MJ kg^−1^) supplemented with pellets containing straw, cereal, hay, clover, barley, oats, lupins, almond shells, oat husks, and limestone (singletons 300 g, twins 500 g; 89% dry matter, metabolizable energy content = 11.6 MJ kg^−1^). Anesthesia was induced with diazepam (0.3 mg kg^−1^, intravenous) followed by ketamine (7 mg kg^−1^, intravenous), then maintained with 1.5% to 2.5% isoflurane in oxygen. Vascular catheters were implanted into the maternal jugular vein, fetal femoral artery and vein as well as the amniotic cavity as described previously (Danielson et al., 2005). Fetal catheters were exteriorized through a small incision in the ewe’s flank. At surgery, antibiotics were administered intramuscularly to the ewe (3.5 mL of 150 mg mL^−1^ procaine penicillin, 112.5 mg mL^−1^ benzathine penicillin, plus 2 mL of 250 g mL^−1^ dihydrostreptomycin) and fetus (1 mL of 150 mg mL^−1^ procaine penicillin, 112.5 mg mL^−1^ benzathine penicillin, plus 1 mL of 250 g mL^−1^ dihydrostreptomycin). Antibiotics were administered intramuscularly to each ewe for 3 days after surgery and intra-amniotically (5 mL of 100 mg mL^−1^ ampicillin) for 4 days after surgery.

### Analgesia and clinical monitoring

**Figure 2.**
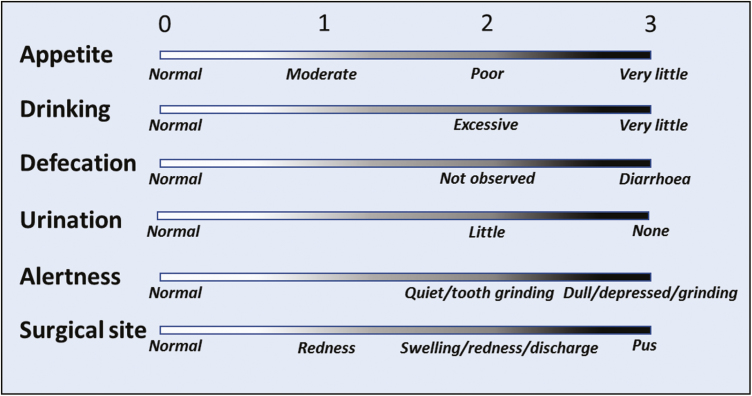
Determination of total clinical score as developed with the SAHMRI Animal Ethics Committee. Ewes are assessed daily by a trained animal technician and scored on a scale of 0 to 3 for each of the indicators as described to generate a total clinical score. Scores >2 require escalation of monitoring and care, with increased monitoring frequency routinely conducted for at least 24 hr following surgery.

Ewes were administered xylazine (20 µg kg^−1^, intramuscular, *n* = 50) upon recovery from anesthesia, or meloxicam (0.5 mg kg^−1^, subcutaneous, *n* = 56) at 1600 hr on both the day prior to and day of surgery. Daily food consumption was determined by weighing daily food offered and residual feed. Routine clinical records were collected for 3 d prior and up to 6 d following surgery by trained animal technicians who were not members of the research group. Ewes were assessed each morning and scored on a scale of 0 to 3 for appetite, drinking, defecation, urination, alertness, and surgical site, where 0 is normal and 3 is severely affected (Figure 2). A total clinical score was calculated as the sum of all scores for each animal and used to determine the appropriate level of monitoring and care as follows: score of ≥2, twice daily monitoring; ≥4, report to principle investigator and animal welfare officer; ≥6, seek veterinary assessment and treat as advised; and ≥10, humane euthanasia followed by post mortem. Total clinical scores and food consumption were analyzed by a linear mixed model to assess effects of treatment (xylazine and meloxicam) and time (day postsurgery) as a repeated measure, with Bonferroni correction for post hoc comparisons at each postsurgical day (IBM SPSS Statistics for Windows, Version 25, Armonk, NY).

## Results

**Figure 3.**
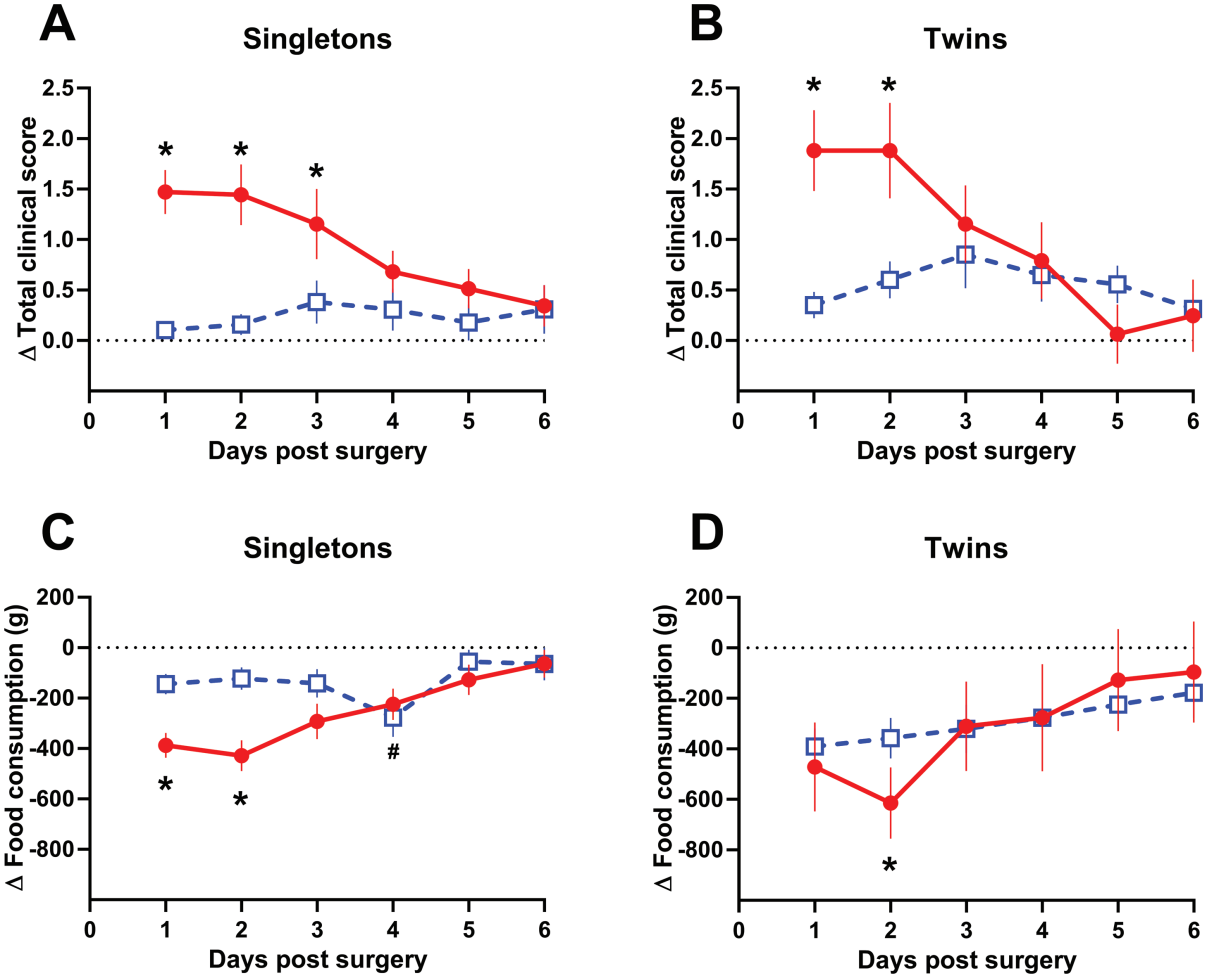
Change in total clinical record score (A and B) and food consumption (C and D) compared with presurgical measures following fetal indwelling catheter surgery in ewes administered xylazine (20 µg kg^−1^; given as a single postoperative dose; red circles; singleton, *n* = 36; twin, *n* = 14) or meloxicam (0.5 mg kg^−1^; given preoperatively 16 to 24 hr prior and at surgery; blue squares; singleton, *n* = 36; twin, *n* = 20). Significant differences from baseline are shown by * in xylazine administered ewes, and # in meloxicam administered ewes (*P* < 0.05).

The effect of surgery on total clinical scores in singleton- and twin-bearing ewes depended on the type of analgesia used (surgery vs. analgesic interaction: singleton *P* = 0.003, twin *P* < 0.001). In singleton-bearing ewes administered xylazine, surgery increased total score at day 1 (1.5 ± 0.4, *P* = 0.001), day 2 (1.4 ± 0.4, *P* = 0.001), and day 3 (1.2 ± 0.4, *P* = 0.036) postsurgery (Figure 3A). In twin-bearing ewes administered xylazine, surgery increased total score at day 1 (1.7 ± 0.4, *P* = 0.001) and day 2 (1.8 ± 0.4, *P* = 0.001) postsurgery (Figure 3B). However, surgery did not alter total clinical scores in either singleton- or twin-bearing ewes administered meloxicam (singleton *P* = 0.616, twin *P* = 0.095).

Similarly, the effect of surgery on food consumption in singleton- and twin-bearing ewes depended on the type of analgesia (surgery vs. analgesic interaction: singleton *P* = 0.04, twin *P* < 0.001). In singleton-bearing ewes administered xylazine, surgery reduced food consumption at day 1 (−387 ± 91 g, *P* = 0.001), day 2 (−403 ± 93 g, *P* < 0.001), and day 3 (−293 ± 91 g, *P* = 0.034) postsurgery (Figure 3C). In twin-bearing ewes administered xylazine, surgery reduced food consumption at day 2 (−536 ± 165 g, *P* = 0.034) postsurgery (Figure 3D). In comparison, postsurgery food consumption in ewes administered meloxicam was significantly reduced only at day 4 (−276 ± 77 g, *P* = 0.008) and only in singleton-bearing ewes.

These results demonstrate that with our previous analgesic regimen of postsurgical xylazine administration, ewes experienced an increase in total clinical scores and reduced appetite for up to 3 d postsurgery. In contrast, these clinical indicators were largely unchanged in ewes administered meloxicam. While this suggests meloxicam provides superior analgesic protection to ewes following fetal indwelling catheter surgery, an analysis of quantifiable parameters that directly assess severity of pain, for example, the Sheep Grimace Scale (Häger et al., 2017), and plasma cortisol as a marker of the physiological stress response are required to confirm this.

## Into the Future

In this review, we have discussed the effectiveness and possible complications of analgesics commonly used in sheep fetal catheterization studies. The primary consideration when choosing an analgesic regimen should be effectiveness. Largely this has involved assessment of maternal behavioral and physiological responses to painful stimuli, and the ability of analgesia to mitigate the response. Fetal perceptions of catheterization should also be considered, and whether systemic maternal administration is likely to have any benefit for the fetus. However, unlike the ewe, it is not possible to accurately or directly assess the impact of analgesia on the fetus. Without the surgically implanted monitoring lines, assessment of fetal condition relies on indirect measures (e.g., ultrasound) taken at a point in time that may not be representative or relevant. Nevertheless, considerations such as route of administration, transport across the placenta, and placenta drug metabolism may influence decisions.

Specific analgesic drugs or drug classes may be more or less appropriate depending on the main outcomes of interest. Fetal catheterization studies cannot be ethically or scientifically justified if the analgesic regimen compromises scientific outcomes. Furthermore, the choice of an appropriate analgesic regimen for specific studies will need to consider issues such as comparability with previous work and the need to maintain consistency throughout an experimental series, to avoid the ethical impact of additional animals needed to repeat previous work. Ideally, factors influencing analgesic choice will be considered and changes implemented at the start of a new series of studies. In addition, restrictions on access to, or the personnel allowed to administer, some classes of licensed drugs, may restrict available options.

Finally, critical to any studies involving maternal and fetal surgery is the need to report the analgesic regimen including dose, route of administration and regimen, as prescribed by the ARRIVE guidelines (Kilkenny et al., 2010) and required by some journals (Grundy, 2015).

## Acknowledgments

JLM was funded by a National Health and Medical Research Council (NHMRC) Career Development Fellowship (2014-2017; APP1066916) and an Australian Research Council Future Fellowship (2018-2021; Level 3; FT170100431). The animal component of the study was funded over several years and thus grants including National Health and Medical Research Council (2012-2014; APP1030853), Canadian Institute of Health Research (2016-2019; PJT-148712), National Heart Foundation (2012-2013; G 11 A 5983) and University of South Australia Research Themes Investment Scheme (2016).

## About the Authors

**Tamara Varcoe** graduated with a Bachelor of Health Sciences (Honours) and then PhD from the Department of Obstetrics and Gynaecology at the University of Adelaide. She conducted postdoctoral research at the Robinson Research Institute of South Australia examining the impact of circadian rhythm disruption on reproductive, metabolic, and neurobehavioral systems. She now works in the Early Origins of Adult Health Research Group at the University of South Australia where she investigates the impact of maternal shift work and sleep disruption upon pregnancy outcomes and long-term health of progeny.

**Jack Darby** is a final year PhD student under the supervision of Professor Janna Morrison in the Early Origins of Adult Health Research Group at the University of South Australia. Jack’s work in this group aims at understanding the fetal programming of adult-onset cardiovascular disease. Specifically, his work focusses on teasing apart the impact of fetal hypoxaemia and hypoglycaemia on cardiac development.

**Kathryn L. Gatford** leads the Early Origins of Adult Health & Disease Research Group in the Robinson Research Institute at the University of Adelaide. She graduated with a Bachelor of Agricultural Science (Honours, dux), and then PhD in animal science at the University of Melbourne. She then moved to the University of Adelaide where she has applied her expertise and interests in animal physiology and endocrinology to studies of pregnancy and developmental programming. She now combines teaching and program coordination with her research, which focuses on effects of perinatal exposures on pregnancy and progeny health and development of interventions to improve long-term outcomes.

**Stacey Holman** is a Research Assistant in the Early Origins of Adult Health Research Group at the University of South Australia. She graduated with a Bachelor of Health Science with Honours at the University of Adelaide and has over 15 years of experience working in scientific research laboratories. Her research interests center on understanding the mechanisms of normal and abnormal development in the fetus to improve the health of mothers and their babies.

**Pearl Cheung** completed a research elective in the Early Origins of Adult Health Research Group as part of her Bachelor of Pharmaceutical Sciences program. She is now completing an internship after completing her Bachelors of Pharmacy and Pharmaceutical Sciences.

**Max Berry** is a Neonatologist, Senior Lecturer in Paediatrics and Director of the Centre for Translational Physiology at the University of Otago. Dr Berry’s research interests include the impact of preterm birth on cardiac autonomic function, vascular function, and development of regional adiposity. In particular, Dr Berry has an interest in translational biomedical research, and the integration of basic sciences with advances in perinatal care.

**Michael Wiese** is an Associate Professor in Pharmacotherapeutics at the University of South Australia School of Pharmacy and Medical Science. He received a Bachelor of Pharmacy, Master of Clinical Pharmacy, and PhD from the University of South Australia, and has worked as clinical pharmacist for 12 yr at The Queen Elizabeth Hospital, Repatriation General Hospital and Royal Hobart Hospital prior to taking up his current position at the University of South Australia. His research interests include the use of pharmacokinetic and pharmacodynamic markers to develop personalized treatment strategies for a number of medical conditions.

**Janna Morrison** is Head of the Early Origins of Adult Health Research Group at the University of South Australia and a Fellow of the Cardiovascular Section of the American Physiological Society. She received her Masters from Western University and her PhD from the University of British Columbia and completed postdoctoral training at the University of Toronto and the University of Adelaide. She currently holds an Australian Research Council Future Fellowship and previously held fellowships from the Heart Foundation of Australia (2004–13) and National Health and Medical Research Council (2014–17). Her current research focusses on how the fetal cardiovascular system responds to changes in nutrient supply during pregnancy.
